# Tumor microenvironment assessment-based signatures for predicting response to immunotherapy in non-small cell lung cancer

**DOI:** 10.1016/j.isci.2024.111340

**Published:** 2024-11-13

**Authors:** Jiani Wu, Yuanyuan Wang, Zhenhua Huang, Jingjing Wu, Huiying Sun, Rui Zhou, Wenjun Qiu, Zilan Ye, Yiran Fang, Xiatong Huang, Jianhua Wu, Jianping Bin, Yulin Liao, Min Shi, Jiguang Wang, Wangjun Liao, Dongqiang Zeng

**Affiliations:** 1 Department of Oncology, Nanfang Hospital, Southern Medical University, Guangzhou, Guangdong 510515, P.R. China; 2 Cancer Center, the Sixth Affiliated Hospital, School of Medicine, South China University of Technology, Foshan, Guangdong 528200, P.R. China; 3 Foshan Key Laboratory of Translational Medicine in Cancer, the Sixth Affiliated Hospital, School of Medicine, South China University of Technology, Foshan, Guangdong 528200, P.R. China; 4 Department of Colorectal Surgery, Shanghai Cancer Center, Fudan University, Shanghai 200032, P.R. China; 5 Department of Cardiology, State Key Laboratory of Organ Failure Research, Nanfang Hospital, Southern Medical University, Guangzhou, Guangdong 510515, P.R. China; 6 Department of Chemical and Biological Engineering, Division of Life Science and State Key Laboratory of Molecular Neuroscience, The Hong Kong University of Science and Technology, Hong Kong, SAR 999077, P.R. China; 7 HKUST Shenzhen-Hong Kong Collaborative Innovation Research Institute, Futian, Shenzhen 518000, P. R. China; 8 Hong Kong Center for Neurodegenerative Diseases, InnoHK, Hong Kong, SAR, P.R. China

**Keywords:** Bioinformatics, Cancer, Omics

## Abstract

Immunotherapy has significantly altered the treatment paradigm of non-small cell lung cancer (NSCLC), but not all patients experience durable benefits. Predictive biomarkers are needed to identify patients who may benefit from immunotherapy. We retrospectively collected tumor tissues from 65 patients with advanced NSCLC before treatment, and performed transcriptomic and genomic analysis. By performing single-sample gene set enrichment analysis, we constructed a predictor named IKCscore based on the tumor microenvironment characteristics. IKCscore is a robust biomarker predicting response to immunotherapy, and its predictive capacity was confirmed from public datasets across different cancer types (*N* = 892), including OAK, POPLAR, IMvigor210, GSE135222, GSE126044, and Kim cohorts. High IKCscore was characterized by inflammatory tumor microenvironment phenotype and higher T cell receptor diversity. The IKCscore exhibits promise as a bioindicator that can predict the efficacy of both immunotherapy and immunotherapy-based combination therapies, while providing guidance for personalized therapeutic strategies for advanced NSCLC patients.

## Highlights

• IKCscore, derived from tumor microenvironment traits, predicts immunotherapy efficacy in NSCLC
• High IKCscore links to inflamed TME and enhaanced T cell receptor diversity
• IKCscore surpasses PD-L1 and TMB in predicting NSCLC response to immunotherapy

## Graphical abstract

**Figure:**
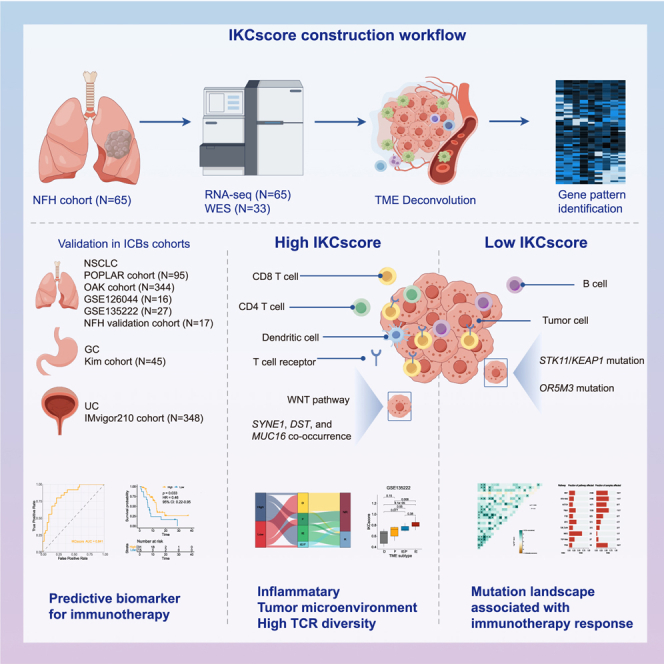

## Introduction

Over the past decade, the treatment of non-small cell lung cancer (NSCLC) has shifted from traditional chemotherapy, radiotherapy, and targeted therapies to immunotherapy. An updated analysis by KEYNOTE-024 revealed that the 5-year survival rate of NSCLC patients treated with immunotherapy increased to 30%,^1^ providing further evidence for using anti-PD-(L)1 antibody in NSCLC. However, a significant subset of patients fails to respond to immune checkpoint blockades (ICBs), with an objective response rate of 20–40%.^2^^,^^3^ Identifying patients who can benefit from ICBs is a significant challenge and forms the basis of precision medicine.

Previous studies on immunotherapy biomarkers mainly focused on PD-L1 immunohistochemistry (IHC), tumor mutation burden (TMB), and microsatellite instability-high (MSI-H) but failed to identify potential responders accurately. PD-L1 expression in tumor cells examined by IHC is the only approved biomarker for determining the response to ICBs in NSCLC and is widely used in clinical practice.^4^ Nevertheless, the use of PD-L1 expression remains controversial because of the non-uniform PD-L1 detection methods and antibodies.^5^ Intriguingly, some patients with negative PD-L1 expression also response to ICBs.^6^ High TMB, which reflects more tumor mutations, is a promising biomarker that generally corresponds to higher immunogenicity and increased T cell reactivity.^7^ However, a significant limitation of TMB is that it does not always correlate with better response to ICBs and prolonged overall survival.^8^^,^^9^ Furthermore, different testing algorithms and unvalidated cut-off values complicate TMB estimation.^10^ Although MSI-H is a pan-tumor immunotherapy predictor, it only occurs in <1% of patients with NSCLC,^11^ suggesting that MSI-H cannot discriminate most candidate responders.

With the rapid development of high-throughput sequencing technology, many tumor microenvironment assessment tools and efficacy biomarkers generated from RNA-seq data have been suggested.^12^ Many computational tools have improved the comprehensive understanding of immune cell infiltration proportion inference, further realizing large-scale tumor microenvironment evaluation based on bulk tumor transcriptome profiles.^13^^,^^14^^,^^15^ Transcriptomic studies have established numerous biomarkers that predict immunotherapy efficacy across multiple cancer types, and several biomarkers are available for clinical applications.^16^^,^^17^ For instance, a T cell-inflamed gene expression profile (GEP) consisting of IFN-γ-related genes showed good predictive power in melanoma.^18^^,^^19^ Recently, we developed a tumor microenvironment score (TMEscore) that predicted ICBs responses with high accuracy^20^ and integrated the TMEscore with a NanoString RNA panel for convenient clinical translation.^21^ In contrast to PD-L1 and TMB, GEP and TMEscore were calculated based on high-throughput data and reflected the multifaceted characterization of tumor and its microenvironment. Therefore, we aimed to develop a reliable biomarker based on pre-treatment NSCLC immunotherapy datasets of RNA-seq data by integrating the tumor microenvironment, intrinsic tumor pathways, and other biological signatures associated with immune responses.

This study utilized the RNA-seq data from NSCLC patients receiving ICBs treatment to construct a robust immune biomarker, the Immune-Keratin-Immune Checkpoint score (IKCscore), based on the tumor microenvironment, and integrated it as an open-source R package for further application in clinical implementation. Furthermore, the potential mechanisms associated with IKCscore were explored in NSCLC, metastatic urothelial cancer, gastric cancer immunotherapy cohorts, and The Cancer Genome Atlas (TCGA) datasets.

## Results

### The establishment of IKCscore

**Figure 1:**
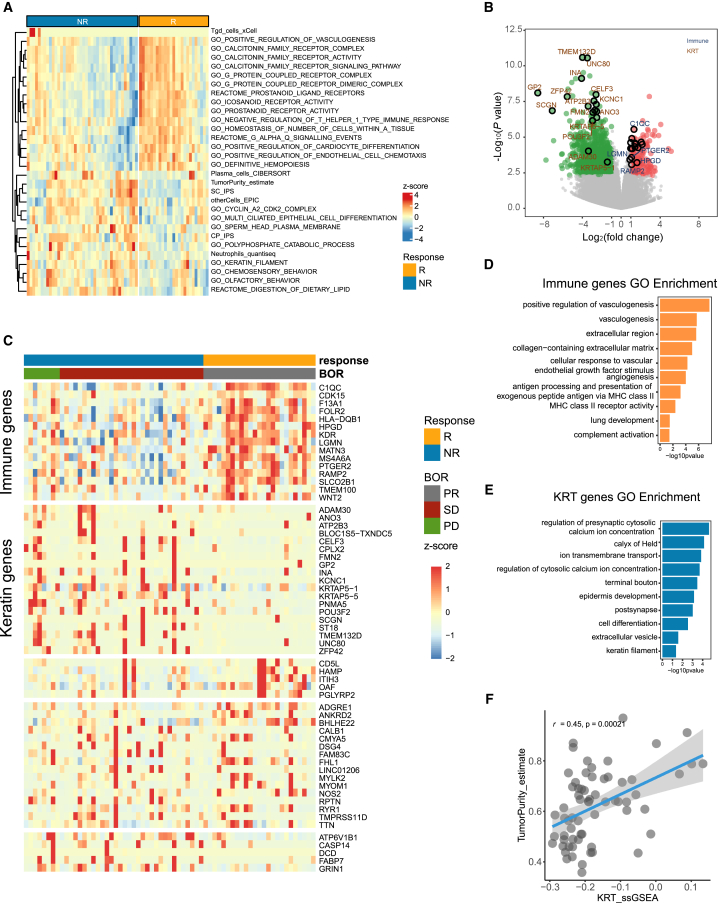
Tumor microenvironment is associated with immunotherapy efficacy in NSCLC (A) The heatmap of the top 15 tumor and tumor microenvironment-associated signature values in responders (R) and non-responders (NR) in the NFH cohort. (B) The volcano plot of differential expression gene analysis between responders (R) and non-responders (NR) in the NFH cohort. The red dots represent significantly upregulated genes (log2Fold Change >1, *p*-value <0.005), and the green dots represent significantly downregulated genes (log2Fold Change <−1, *p*-value <0.005). The represented genes in IKCscore were marked with a black circle. (C) Unsupervised K-means clustering of selected immunotherapy efficacy-associated genes. (D) Gene ontology enrichment analysis of genes in Immune pattern. (E) Gene ontology enrichment analysis of genes in KRT pattern. (F) A scatterplot demonstrated a positive correlation between the TumorPurity and KRTscore (Spearman test, r = 0.45, *p* = 0.00021).

Transcriptome sequencing was performed on tumor specimens from 65 patients with advanced NSCLC before receiving anti-PD-1 therapy in our Nanfang Hospital (NFH) cohort. The baseline clinicopathological features of the patients are summarized in Table 1; detailed information is provided in Table S1. The IKCscore establishment flowchart was shown in Figure S1. We first calculated numerous signatures covering tumor microenvironment, metabolic pathways, tumor intrinsic pathways via R package IOBR.^22^ Subsequently, batch Wilcoxon statistical analyses were used to select the signatures associated with immunotherapy responses. And the top 15 signatures highly expressed in responders (complete response (CR)/partial response (PR)) and non-responders (progressive disease (PD)/stable disease (SD)) ordered by *p*-value (*p* < 0.05) respectively were identified (Figure 1A). Next, we reviewed the gene sets of these 30 signatures (Figure 1A), listing the component genes (*n* = 280). For these 280 genes, we applied the Wilcoxon test to screen for genes significantly correlated with immunotherapy responses (responders vs. non-responders, *p* < 0.05) and obtained 16 genes. Simultaneously, by performing differential gene expression (DEG) analysis, we identified genes that were significantly upregulated in responders and non-responders (Figure 1B), and the top 35 genes ordered by *p*-value in both responders and non-responders respectively were selected. Next, so as to further integrate the genes associated with therapeutic responses from the above steps, we combined 16 genes that constitute the response-associated signatures and 70 genes identified from DEGs, and got 86 genes. Further, we removed all pseudogenes and obtained 79 genes at last. To readily facilitate clinical application, we screened the top 30 significant genes related to ICBs efficacy respectively in responders and non-responders from the 79 genes again to further reduce gene numbers using Wilcoxon statistical analyses (*p* < 0.05). Finally, we obtained 60 genes associated with responses to immunotherapy. Next, K-means were utilized to identify gene patterns relevant to optimal immunotherapy response on 60 selected genes. The K-means cluster analysis of selected 60 genes generated 5 gene expression patterns (Figure 1C). Interestingly, the first pattern of genes was highly expressed in responders to ICBs, and Gene Ontology (GO) enrichment analysis demonstrated that they were predominantly enriched in the immune-associated pathways including antigen processing and presentation, MHC class receptor activity, and complement activation (Figure 1D). Therefore, the first cluster was defined as immune pattern. Notably, the second pattern genes highly expressed in non-responders to ICBs. GO enrichment analysis demonstrated that the second pattern genes were enriched in epidermal development and keratin filament pathways, which were commonly correlated with tumor development^23^^,^^24^ (Figure 1E). Therefore, the second pattern was defined as the Keratin pattern. Then, we sought to use the single-sample Gene Set Enrichment Analysis (ssGSEA) algorithm to derive signature score characterizing immune pattern genes and Keratin pattern genes separately. Consistently, a positive correlation was observed between TumorPurity and Keratin score (KRTscore) (Figure 1F), further confirming that KRTscore was a negative indicator for immunotherapy efficacy. Since immune checkpoints are the targets of immunotherapy, we aim to integrate immune checkpoint related genes to further improve the accuracy of immunotherapy efficacy prediction. Mariathasan et al. collected several immune-associated gene sets, including the Immune Checkpoint gene set,^25^ which contained PDL1, PDCD1LG2, CTLA4, PDCD1, LAG3, HAVCR2, and TIGIT. By applying the ssGSEA, we obtained the signature score reflecting the above immune checkpoint genes expressions, named Immune Checkpoint score. In general, we used positive response-related signature scores, including Immune score and Immune Checkpoint score, and subtracted the negatively related signature score KRTscore to generate an integrated model named IKCscore for predicting the immunotherapy responses: IKCscore = Immune score + Immune Checkpoint score – KRTscore. The component genes of Immune pattern and Keratin pattern are listed in Table S2.

**Table 1:** Baseline characteristics of patients with advanced non-small cell lung cancer in NFH cohort

	Total (*N* = 65)
Age	59.07 (31–81)
Sex	Male (52, 80%)
	Female (13, 20%)
Race	Asian (65, 100%)
Histology	Adenocarcinoma (36, 55%)
	Squamous carcinoma (20, 31%)
	Others (9, 14%)
Regimen	Monotherapy (14, 22%)
	Combination (51, 78%)
Best objective response	PR (25, 39%)
	SD (32, 49%)
	PD (8, 12%)

### IKCscore is a promising predictor of immunotherapy response in NSCLC

**Figure 2:**
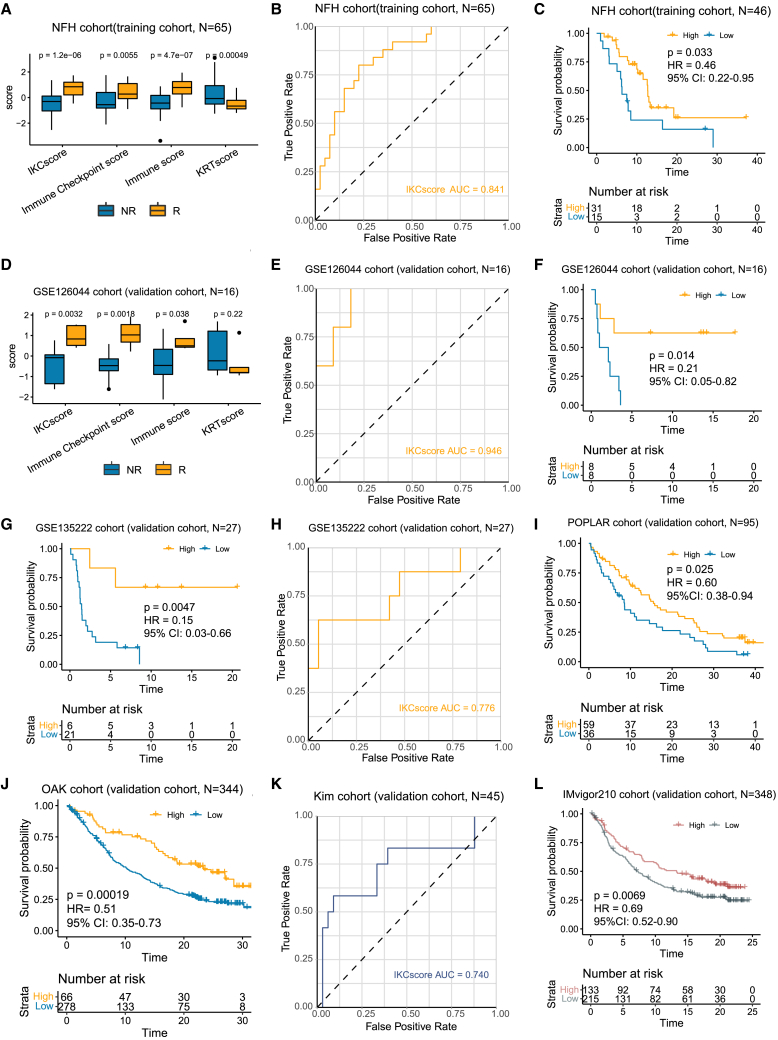
IKCscore holds promise in predicting immunotherapeutic response (A) Boxplot showed that increased IKCscore, Immune Checkpoint score, and Immune score in the R group and an increased KRTscore in the NR group in the NFH cohort (NFH cohort: *n* = 65; Wilcoxon test, *p* = 1.2e-06, 0.0055, 4.7e-07, 0.00049, respectively). (B) ROC analysis indicated that the IKCscore achieved an AUC of 0.841 in the NFH cohort. (C) High IKCscore was significantly related to more favorable PFS in the NFH cohort (Log rank, *p* = 0.033, HR = 0.46, 95% CI: 0.22–0.95). (D) Boxplot showed increased IKCscore, Immune Checkpoint score, and Immune score in the R group and increased KRTscore in the NR group in the GSE126044 cohort (GSE126044 cohort: *n* = 16; Wilcoxon test, *p* = 0.0032, 0.0018, 0.038, 0.22, respectively). (E) ROC curve of IKCscore in GSE126044 cohort (AUC = 0.946). (F) Kaplan-Meier survival analysis demonstrated that a higher IKCscore was significantly related to more favorable PFS in the GSE126044 cohort (Log rank, *p* = 0.014, HR = 0.21, 95% CI: 0.05–0.82). (G) Kaplan Meier survival analysis showed that patients with high IKCscore had significantly longer PFS in the GSE135222 cohort (GSE135222 cohort: *n* = 27; Log rank, *p* = 0.0047, HR = 0.15, 95% CI: 0.03–0.66). (H) ROC curve of IKCscore in GSE135222 cohort. (I and J) Kaplan-Meier survival analysis revealed that a high IKCscore was significantly correlated with prolonged OS in the (I) POPLAR cohort (*n* = 95) and (J) OAK cohort (*n* = 344) (Log rank, POPLAR: *p* = 0.025, HR = 0.60, 95% CI: 0.38–0.94; OAK: *p* = 0.00019, HR = 0.51, 95% CI: 0.35–0.73). (K) ROC curve of IKCscore in metastatic gastric cancer cohort (*n* = 45, AUC = 0.740). (L) Kaplan-Meier analysis of overall survival in the IMvigor210 cohort (*n* = 348, Log rank, *p* = 0.0069, HR = 0.69, 95% CI: 0.52–0.90), with patients divided by high and low IKCscore. R, responders; NR, non-responders; AUC, Area under curve; ROC, Receiver operating characteristic.

In the NFH cohort, the relationship between the IKCscore and treatment responses was evaluated. Consistent with our expectations, the Immune score, Immune Checkpoint score, and IKCscore were associated with therapeutic response, whereas the KRTscore was significantly increased in the non-responder group (Figure 2A). Receiver operating characteristic (ROC) curve analysis revealed that IKCscore achieved an area under curve (AUC) value of 0.841, suggesting that IKCscore had high accuracy in predicting ICBs responses (Figure 2B). Additionally, compared to patients with low IKCscore, those with high IKCscore had significantly improved progression-free survival (PFS) (Figure 2C, HR = 0.46, 95% CI:0.22–0.95, *p* = 0.033). We further validated the predictive capacity of the IKCscore in other NSCLC immunotherapy datasets. In the GSE126044 cohort and GSE135222 cohort, the predictive value of the IKCscore for immunotherapy was confirmed (GSE126044: Figures 2D and 2E, IKCscore AUC = 0.946; Figure 2F, HR = 0.21, 95% CI:0.05–0.82, *p* = 0.014; GSE135222; Figure 2G, HR = 0.15, 95% CI:0.03–0.66, *p* = 0.0047; Figure 2H, IKCscore AUC = 0.776). In addition, patients with high IKCscore had significantly longer overall survival than those with low IKCscore in the POPLAR and OAK cohort (Figure 2I, HR = 0.60, 95% CI:0.38–0.94, *p* = 0.025; Figure 2J, HR = 0.51, 95% CI:0.35–0.73, *p* = 0.00019), suggesting that patients with high IKCscore had a favorable response to ICBs.

To expand the applicability of the IKCscore to other cancer types, we investigated the performance of the IKCscore in advanced gastric cancer and metastatic urothelial cancer. As expected, the IKCscore exhibited good predictive capacity for anti-PD-1 therapy in patients with gastric cancer (Figure 2K, AUC = 0.740). Survival analysis also revealed that patients with high IKCscore had prolonged PFS in the IMvigor210 cohort (Figure 2L, HR = 0.69, 95% CI:0.52–0.90, *p* = 0.0069). Accordingly, our study showed that the IKCscore might be a robust biomarker for predicting ICBs responses in NSCLC.

### Comparison of IKCscore and PD-L1 in predicting immunotherapy monotherapy and combined therapy efficacy

**Figure 3:**
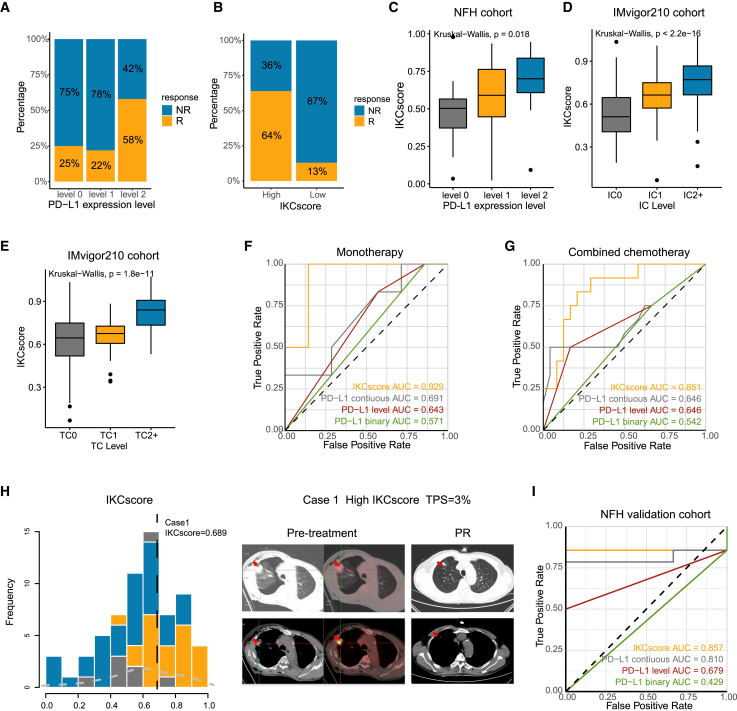
Comparison of IKCscore and PD-L1 in predicting immunotherapy monotherapy and combined therapy (A) Rate of response to ICBs in different PD-L1 expression groups, including level 0, level 1, and level 2 in the NFH cohort. PD-L1 level 0: <1%; level 1: 1–49%; level 2: ≥50%. (B) Rate of response to ICBs in high and low IKCscore groups in the NFH cohort. (C) IKCscore was positively associated with PD-L1 expression level (Kruskal−Wallis, *p* = 0.018). (D and E) In IMvigor210 study, tumor tissue samples were scored through immunohistochemistry (IHC) for PD-L1 expression on tumor-infiltrating immune cells (IC), which included macrophages, dendritic cells and lymphocytes. Specimens were scored as IHC IC0, IC1, IC2, or IC3 if <1%, ≥1% but <5%, ≥5% but <10%, or ≥10% of IC were PD-L1 positive, respectively. The PD-L1 expression on tumor cells (TC) was also conducted. Specimens were scored as IHC TC0, TC1, TC2, or TC3 if <1%, ≥1% but <5%, ≥5% but <50%, or ≥50% of TC were PD-L1 positive, respectively. PD-L1 expression, both IC (D) and TC (E), are positively correlated with IKCscore in the IMvigor210 cohort. IC0 and TC0 tumors have the lowest IKCscore compared to IC1, IC2+, TC1, and TC2+ (IC: Kruskal−Wallis, *p* < 2.2e−16; TC: Kruskal−Wallis, *p* = 1.8e−11). (F) ROC curve of IKCscore, continuous PD-L1 expression, PD-L1 level, and PD-L1 binary in NFH cohort with PD-L1 IHC results who received anti-PD-1 inhibitor monotherapy (AUC = 0.929, 0.691, 0.643, 0.571, respectively). (G) ROC curve of IKCscore, continued PD-L1 expression, PD-L1 level, and PD-L1 binary in NFH cohort with PD-L1 IHC results who received anti-PD-1 inhibitor plus chemotherapy combination therapy (AUC = 0.851, 0.646, 0.646, 0.542, respectively). (H) The IKCscore distribution of case 1 patient and the typical CT image at the baseline and PR times. (I) ROC curve of IKCscore, continuous PD-L1 expression, PD-L1 level, and PD-L1 binary in NFH validation cohort receiving anti-PD-1 inhibitor plus chemotherapy combination therapy (AUC = 0.857, 0.810, 0.679, 0.429, respectively).

PD-L1 expression remains the only reliable biomarker of immunotherapy in advanced NSCLC and is widely used in clinical practice. To describe the relationship between the IKCscore and PD-L1 expression, we stratified PD-L1 expression into three levels: <1% (level 0), 1–49% (level 1), and ≥50% (level 2). Consistent with our expectations, 58% of patients with level 2 PD-L1 expression responded to immunotherapy (Figure 3A). In comparison, 25% and 22% of patients with level 0 and 1 PD-L1 expression were responders, respectively, implying that a subset of PD-L1-negative patients still benefit from immunotherapy. Patients with higher IKCscore were more likely to benefit from ICBs therapy (Figure 3B). The boxplot demonstrated that the IKCscore increased with increasing PD-L1 expression levels (Figure 3C, Kruskal−Wallis, *p* = 0.018). In addition, the IKCscore was positively correlated with PD-L1 expression level, including immune cells (IC) and tumor cells (TC) levels in the IMvigor210 cohort (Figures 3D and 3E, IC: Kruskal−Wallis, *p* < 2.2e−16; TC: Kruskal−Wallis, *p* = 1.8e−11).

Next, we sought to compare the predictive abilities of the IKCscore and PD-L1 expression. Continuous PD-L1 expression represents the original IHC results; PD-L1 was classified as binary into positive (≥1%) and negative (<1%) PD-L1 expression; PD-L1 levels are presented as previously mentioned. Therefore, we further analyzed the IKCscore and PD-L1 expression performance in different regimen settings. Among the 49 patients with available PD-L1 IHC results, 13 received ICBs monotherapy, and 36 received ICBs combination therapy. In both monotherapy and combination therapy groups, the IKCscore yielded a higher predictive accuracy (Figure 3F: IKCsocre AUC = 0.929; Figure 3G: IKCscore AUC = 0.851) than PD-L1 expression (Figure 3F: continuous PD-L1 AUC = 0.691, PD-L1 level AUC = 0.643, PD-L1 binary AUC = 0.571; Figure 3G: continuous PD-L1 AUC = 0.646, PD-L1 level AUC = 0.646, PD-L1 binary AUC = 0.542). Case 1 patient owned a high IKCscore but TPS = 3% at baseline. PET-CT was utilized as a complementary measure to evaluate hypermetabolic lesions. After immunotherapy and chemotherapy combination therapy, the response rate was assessed as PR (Figure 3H). The high accuracy of the IKCscore in discriminating responders from combination therapy arouses concerns due to the lack of biomarkers indicating immunotherapy combination therapy efficacy in NSCLC. Further, 17 NSCLC samples undergoing ICBs-based combination therapy were collected for RNA-seq as the validation cohort. In the validation cohort, the IKCscore also exhibited a higher AUC than PD-L1 variables (Figure 3I: IKCscore AUC = 0.857, continuous PD-L1 AUC = 0.810, PD-L1 level AUC = 0.679, PD-L1 binary AUC = 0.429). Collectively, IKCscore could not only screen out responders and non-responders from ICBs monotherapy but also displayed higher accuracy than PD-L1 expression level for ICBs combination therapy.

### Comparisons and associations of TMB and T cell receptors diversity with IKCscore

**Figure 4:**
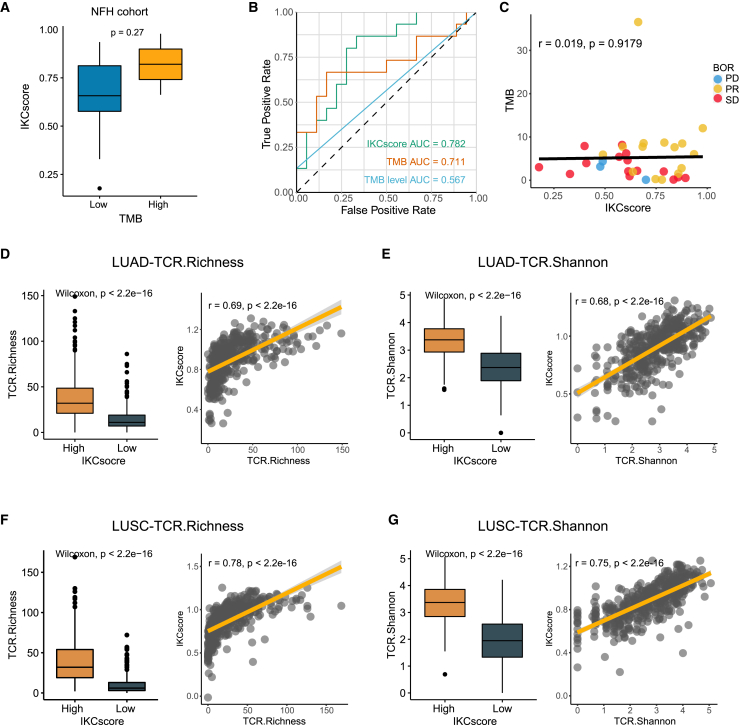
Comparison and associations of TMB and TCR diversity with IKCscore (A) Boxplot depicted IKCscore level in high TMB and low TMB groups (Wilcoxon test, *p* = 0.27). (B) Comparison of ROC curves of IKCscore, continuous TMB, and TMB group (cutoff = 10 mut/Mb) in patients who owned TMB results (AUC = 0.782, 0.711, 0.567, respectively). (C) Scatterplots showed that the IKCscore and TMB were irrelevant in the NFH cohort (Spearman test, r = 0.019, *p* = 0.9179). The dotted color indicates the different responses (PR: yellow; SD: red; PD: blue). (D and E) TCR richness (D) and TCR Shannon (E) was positively associated with IKCscore in TCGA-LUAD (TCR.Richness: Wilcoxon test, *p* < 2.2e−16; Spearman test, r = 0.69, *p* < 2.2e-16; TCR.Shannon: Wilcoxon test, *p* < 2.2e−16; Spearman test, r = 0.68, *p* < 2.2e-16). (F and G) TCR richness (F) and TCR Shannon (G) was positively associated with IKCscore in TCGA-LUSC (TCR.Richness: Wilcoxon test, *p* < 2.2e−16; Spearman test, r = 0.78, *p* < 2.2e-16; TCR.Shannon: Wilcoxon test, *p* < 2.2e−16; Spearman test, r = 0.75, *p* < 2.2e-16). TMB, Tumor mutation burden; TCR, T cell receptors.

Although TMB is a controversial biomarker of ICBs efficacy in NSCLC, its potential in predicting efficacy cannot be underestimated. To explore the relationship between TMB and IKCscore, we categorized binary TMB into high and low TMB, with a cut-off TMB value of 10 (mut/Mb), based on Food and Drug Administration approval.^26^ The IKCscore increased in the high TMB group, but only 2 patients were identified as high TMB (Figure 4A, Wilcoxon test, *p* = 0.27). In the NFH cohort, the IKCscore displayed a higher predictive value than TMB and binary TMB (Figure 4B, AUC = 0.782, 0.711, and 0.567, respectively). Correlation analysis also confirmed that the IKCscore and TMB were not correlated in the NFH cohort (Figure 4C, r = 0.019, *p* = 0.9179), implying that the IKCscore and TMB-mediated ICB responses occur via distinct mechanisms.

The tumor mutation burden identifies neoantigens derived from nonsynonymous mutations.^10^ Neoantigens are processed and presented and can then be captured by specific T cell receptors (TCR), further activating the host anti-tumor response.^27^ Therefore, TCR diversity representing identified neoantigens appears to be a more accurate predictor of tumor response to therapy than TMB diversity. In melanoma, high TCR diversity before treatment was associated with anti-PD1 therapy responses.^28^ High TCR diversity also reflects activated immune status in lung cancer, which could indicate anti-cancer treatment efficacy.^29^ Consistently, in The Cancer Genome Atlas Lung Adenocarcinoma (TCGA-LUAD) and The Cancer Genome Atlas Lung Squamous Cell Carcinoma (TCGA-LUSC), the IKCscore was positively correlated with TCR diversity, including TCR Shannon and TCR richness (Figures 4D–4G), indicating that the IKCscore is capable of identifying patients with high TCR diversity who probably benefit from immunotherapy.

### IKCscore is associated with the immune-activated microenvironment

**Figure 5:**
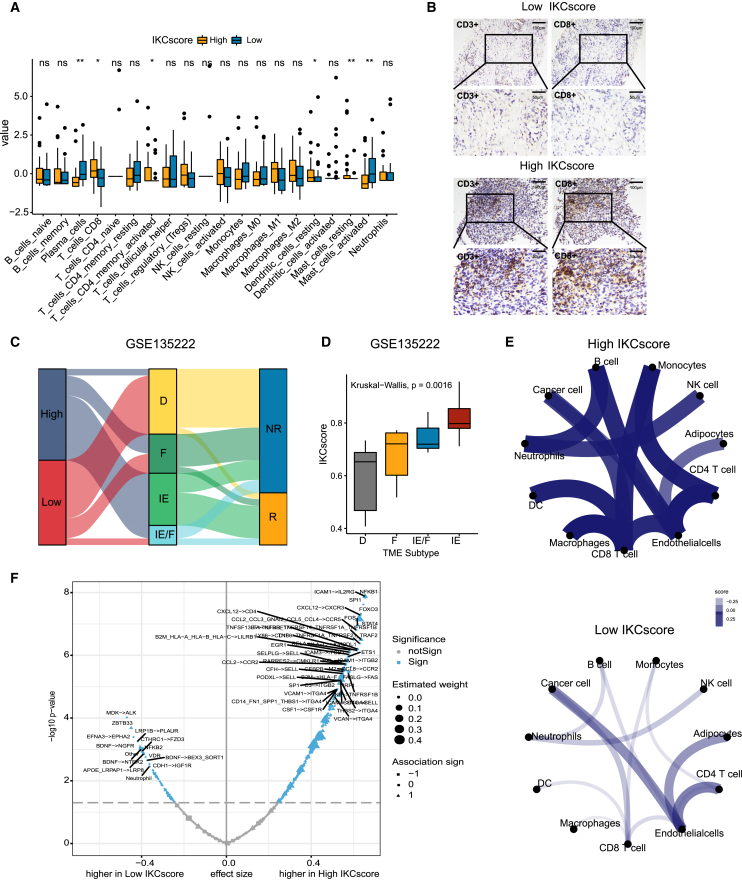
IKCscore is associated with the immune-activated microenvironment (A) CIBERSORT calculated the fraction of immune cells in the high and low IKCscore groups. The statistical difference between the two groups was compared through the Wilcoxon test. ∗, *p* < 0.05; ∗∗, *p* < 0.01; ns, not significant. (B) Representative images of immunohistochemical stainings of CD3 and CD8 for resected tumor specimens in high IKCscore and low IKCscore. (C) Alluvial diagram of high and low IKCscore groups with different TME subtypes (D, F, IE, and IE/F) and clinical response in GSE135222 cohort. (D) The boxplot showed that IKCscore mainly increased in the IE subtype, while the lowest IKCscore was observed in the D subtype in the GSE135222 cohort (Kruskal−Wallis, *p* = 0.0016). (E) The cell interaction network in high and low IKCscore groups. (F) The volcano plot revealed the ligand-receptor pairs, transcription factors, and other factors related to IKCscore. IE, immune-enriched, non-fibrotic; IE/F, immune-enriched/fibrotic; F, fibrotic; D, immune-depleted.

To determine whether IKCscore could characterize the immune microenvironment, we assessed the association between IKCscore and immune cell infiltration abundance using CIBERSORT. In the NFH cohort, CD8^+^ T cells, memory resting CD4^+^ T cells, and resting dendritic cell infiltration levels in the high IKCscore group were significantly higher than those in the low IKCscore group (Figure 5A). The infiltration of CD8^+^ T cells was further examined by IHC (Figure 5B). Additionally, CD8^+^ T cell signatures, dendritic cell signatures, and antigen presentation-associated signatures were enriched in the high IKCscore group (Figure S2A). Multiple studies have classified tumor subtypes according to the pattern of immune cell infiltration. In metastatic urothelial cancer (IMvigor210), high IKCscore implied an inflamed subtype linked to a better response (Figures S2B and S2C). Bagaev et al. recently established 4 simple tumor microenvironment (TME) subtypes, termed immune-enriched, fibrotic (IE/F), immune-enriched, non-fibrotic (IE), fibrotic (F), and immune-depleted (D) across 20 cancer types, which characterized the landscape of immune cells and fibrotic cells, and served as a potential immunotherapy indicator.^30^ As expected, high IKCscore with more IE and IE/F subtypes was linked to a better response in the GSE135222 cohort (Figures 5C and 5D). Similarly, the IKCscore increased in the IE and IE/F subtypes in TCGA-LUAD (Figure S2D) and TCGA-LUSC (Figure S2E) cohorts. We then explored the potential interaction between tumor cells and microenvironment cells using the R package EaSIeR.^31^ Complex intercellular communication networks involved with tumor cells and the microenvironment were observed in high IKCscore samples compared with low IKCscore samples, especially interactions between CD8^+^ T cells and other immune cells (Figure 5E). Interestingly, a more complicated network was also observed in the responders to immunotherapy (Figure S2F). In addition, several factors, including TME ligand-receptor pairs, transcription factors, and immune cells related to the IKCscore, were identified, which highly overlapped with those related to immunotherapy response (Figures 5F and S2G). Together, high IKCscore reflected an immune-activated microenvironment where more immune cells infiltrated with more potential to arouse the host anti-tumor response.

### Mutation landscape between high and low IKCscore

**Figure 6:**
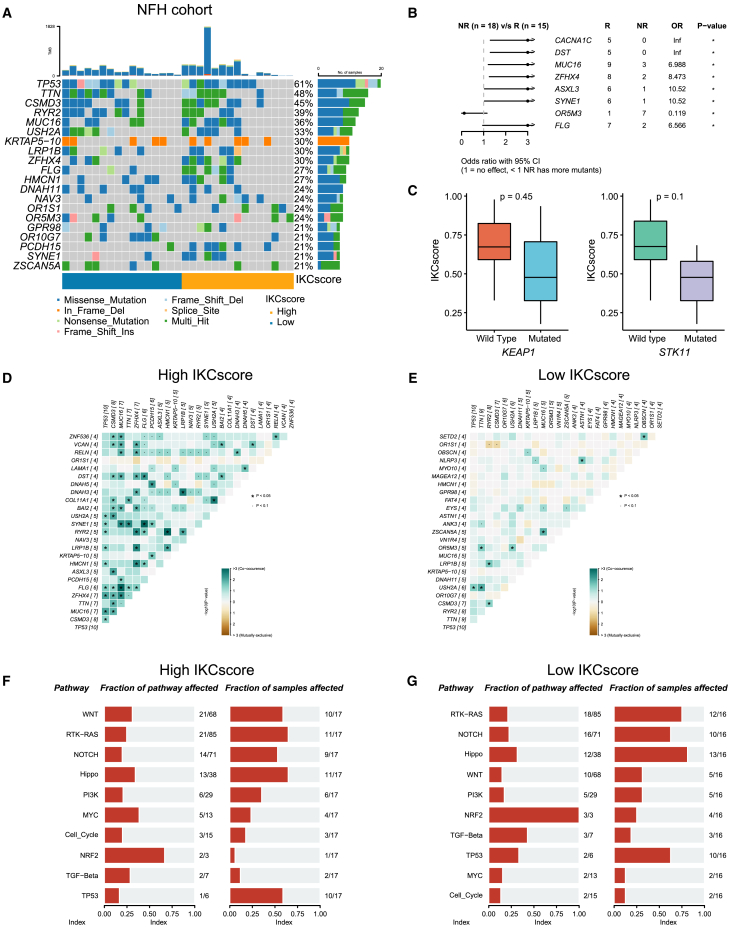
Mutation landscape between high and low IKCscore (A) The oncoplot of the top 20 mutated genes in high and low IKCscore groups in the NFH cohort. (B) Forest plots identified significant differential gene mutations associated with ICBs responses. ∗, *p* < 0.05. (C) KEAP1 and STK11 mutations were significantly correlated with lower IKCscore compared to the wild type (Wilcoxon test, *p* = 0.45, *p* = 0.1, respectively). (D and E) Co-occurrence and co-exclusive analysis of gene mutations in the high IKCscore (D) and low IKCscore (E) groups. (F and G) Mutant pathway enrichment in high IKCscore (F) and low IKCscore (G) groups.

Somatic gene mutations can impact therapeutic efficacy via interaction with tumor microenvironment. We attempted to reveal the genomic determinants of immunotherapy efficacy. Next, we aimed to determine the association between the IKCscore and gene alteration by conducting whole genome sequencing (WES) on tumor tissue samples taken before immunotherapy in 33 patients in the NFH cohort. An oncoplot revealed the frequency of common gene alterations associated with IKCscore in the NFH cohort (Figure 6A), TCGA-LUAD (Figure S3A), and TCGA-LUSC (Figure S3C) cohorts. Response-associated gene mutations were identified using the R package maftools. Consistent with previous studies, *MUC16*^32^ and *SYNE1*^33^ mutations were confirmed to be associated with better immunotherapy responses. Except for the above genes, *CACNA1C*, *DST*, *ZFHX4*, *ASXL3*, and *FLG* mutations were observed in responders to immunotherapy, some of which were also enriched in the high IKCscore group, which might be potential predictors of ICBs. Conversely, the *OR5M3* mutation appeared more frequently in the non-responders (Figure 6B). In addition, as shown in previous studies, *STK11* and *KEAP1* mutations have been recognized as indicators of decreased efficacy of anti-PD-(L)1 treatment in NSCLC. In the NFH cohort, IKCscore downregulation was observed in patients with *KEAP1* and *STK11* mutations (Figure 6C) compared to the wild-type subset, albeit without statistical significance. Further analysis of TCGA-LUAD (Figure S3B) and TCGA-LUSC (Figure S3D) confirmed the relevance of these mutations, which are associated with drug resistance toward ICBs. Correlation analysis revealed that *SYNE1*, *DST*, *FLG*, and *MUC16* were co-occurrence in the high IKCscore group (Figure 6D). In contrast, few co-occurrences were observed in the low IKCscore group (Figure 6E). Intriguingly, the WNT pathway was enriched in the high IKCscore group, indicating that WNT pathway mutations might predict ICBs responses (Figures 6F, 6G, S3E, and S3F). The result of mutation landscape between high and low IKCscore might provide clues for the high accuracy of the IKCscore in predicting ICBs efficacy.

## Discussion

Although immunotherapy has revolutionized the treatment of metastatic NSCLC, only a minority of patients derive durable benefits from anti-PD-1 therapy. Owing to the inherent deficiencies of PD-L1, TMB, and other prevalent predictors,^5^^,^^7^ identifying robust biomarkers is warranted to promote precision oncology. The present study systematically integrated diverse tumor microenvironments and biological signatures associated with immunotherapy responses. We developed the IKCscore as a robust biomarker for predicting immunotherapeutic efficacy in advanced NSCLC, which has also been validated in immunotherapy cohorts. Our results demonstrated that the IKCscore showed higher predictive accuracy than TMB and PD-L1 expression in identifying responders, especially in anti-PD-1 combined with chemotherapy. In addition, tumors with high IKCscore have an inflammatory microenvironment rich in immune cells that are more responsive to anti-tumor immune therapies.

PD-L1 expression, measured by IHC, is still the most widely used clinical predictor of immunotherapy response in advanced NSCLC. However, some patients with low PD-L1 expression may also respond to ICBs therapy and even have prolonged overall survival, especially those receiving ICBs combined with chemotherapy.^34^^,^^35^ For immunotherapy plus chemotherapy, PD-L1 performed poorly in discriminating candidate patients who could benefit from the combination therapy. For instance, in the KEYNOTE-189 trial,^36^ improved overall survival and objective response rates were found in pembrolizumab plus pemetrexed-platinum regimen compared to placebo plus pemetrexed-platinum regimen in patients with metastatic NSCLC, regardless of PD-L1 expression level. Similarly, the KEYNOTE-407 study proved that immunotherapy combined with chemotherapy could prolong survival in advanced-stage NSCLC, including in patients with PD-L1 expression <1%.^37^ Previous clinical trials have indicated that PD-L1 expression is unsuitable for predicting the efficacy of immunotherapy and chemotherapy. TMB also weakly discriminated candidate responders to ICBs combined with chemotherapy. Our cohort analysis confirmed that in the context of ICBs therapy combined with chemotherapy, the IKCscore achieved high predictive accuracy, while PD-L1 expression was not appropriate for patient selection. Hence, the IKCscore was expected to enhance the accuracy of identifying responders to combination treatment.

Notably, the interaction between the TME and intrinsic variables has been identified as a crucial factor affecting immunotherapy responses. CD8^+^ T cell infiltration, IFN-γ, and antigen processing and presentation have been investigated as mechanisms of sensitivity to immunotherapy in NSCLC.^38^^,^^39^^,^^40^^,^^41^ For instance, Gettinger et al. found defective antigen processing and presentation in patient-derived xenografts established from ICB-resistant NSCLC tumors, indicating that antigen processing and presentation disruption could mediate immune escape from ICBs.^39^ In line with this, our results demonstrated that high CD8^+^ T cell and dendritic cell infiltration corresponded with high IKCscore, as well as high antigen processing and presentation-related signature scores, implying a better response to ICBs. TCRs identify tumor cells presenting neoantigens generated from gene mutations and establish anti-tumor activity. In our study, the IKCscore was positively correlated with TCR diversity. Therefore, the accuracy of the IKCscore in predicting immunotherapy efficacy is partly attributed to the fact that it indicates a pre-existing immune state mediated by TCR diversity.

In conclusion, we established a robust predictor, the IKCscore, and verified its predictive ability in independent immunotherapy cohorts. The IKCscore holds promise in identifying responders to immunotherapy combined with chemotherapy in advanced NSCLC surpassing PD-L1 expression; however, prospective studies are warranted.

### Limitations of the study

This study has several limitations. Although the IKCscore exhibited immunotherapy predictive ability in different cancer types, it performed suboptimal in melanoma due to unique biological characterization. Furthermore, there is heterogeneity in the immunotherapy cohorts of different cancer types and different detection methods including RNA-seq and microarray data, making it difficult to define a unified cut-off standard across multiple cohorts. Lastly, the deficiency is that the IKCscore is calculated based on the ssGSEA algorithm, which requires the expression profile of all genes within each dataset and cannot be calculated individually. To conquer this deficiency, we are working to establish a NanoString panel using IKCscore signature genes in prospective study cohort (NCT06232265), which could normalize gene expression using house-keeping genes and estimate IKCscore individually.

## Resource availability

### Lead contact

Further information and requests for resources and reagents should be directed to and will be fulfilled by the lead contact, Dongqiang Zeng (interlaken@smu.edu.cn).

### Materials availability

The study did not generate new unique reagents.

### Data and code availability

• The data for the NFH cohort generated in this study were deposited in the China National Center for Bioinformation, are available upon request from the corresponding author under the accession code ID HRA003748.
• The study incorporated IKCscore methodology into an open-source R package, available on GitHub at https://github.com/LiaoWJLab/IKCscore.
• Any additional information required to reanalyze the data reported in this paper is available from the lead contact upon request.

## Acknowledgments

This work was supported by the 10.13039/501100001809National Natural Science Foundation of China (No. 82073303 to Wangjun Liao), Guangdong Province Science and Technology Plan Project (No. 2020A0505090007 to Jiguang Wang), Science and Technology Projects in Guangzhou (No. 2023A04J2377 to Jiani Wu, and No. 2023A04J2357 to Dognqiang Zeng), and 10.13039/501100002858China Postdoctoral Science Foundation (No. 2023M741580 to Jiani Wu).

## Author contributions

Conceptualization: Jiani Wu (Author 1), W.L., and D.Z.; Data curation: Jiani Wu (Author 1), Y.W., and Z.H.; Formal analysis: Jiani Wu (Author 1), R.Z., and W.Q.; Visualization: Jiani Wu (Author 1), Jingjing Wu (Author 2), R.Z., and D.Z.; Methodology: Jiani Wu (Author 1), Y.F., and D.Z.; Writing–original draft: J.W. and X.H.; Funding acquisition: Jiani Wu (Author 1), Jiguang Wang (Author 4) and W.L.; Resources: Y.W., Z.H., H.S., Jiguang Wang (Author 4), and W.L.; Investigation: Y.W., Z.H., H.S., and Jianhua Wu (Author 3); Project administration: Y.W., Z.H., J.B., Y.L., and M.S.; Software: D.Z.; Supervision: J.B., Y.L., M.S., Jiguang Wang (Author 4), and W.L.; Writing–review and editing: Z.Y. and D.Z.

## Declaration of interests

The authors declare no competing interests.

## STAR★Methods

### Key resources table

**Table:** 

REAGENT or RESOURCE	SOURCE	IDENTIFIER
**Antibodies**		

Anti-CD3 antibody	Abcam	Cat# ab16669, RRID: AB_443425
Anti-CD8 antibody	Abcam	Cat# ab93278, RRID: AB_10563532
Goat Anti-Rabbit IgG	Abbkine	Cat# A21020, RRID: AB_2876889

**Deposited data**		

RNA-seq, genomic data and clinical data for the TCGA cohort	TCGA	https://xenabrowser.net/datapages
The TCR diversity profiling data	Bagaev et al. study	https://doi.org/10.1016/j.ccell.2021.04.014
GSE135222	Gene Expression Omnibus (GEO)	https://www.ncbi.nlm.nih.gov/geo/query/acc.cgi?acc=GSE135222
GSE126044	Gene Expression Omnibus (GEO)	https://www.ncbi.nlm.nih.gov/geo/query/acc.cgi?acc=GSE126044
IMvigor210	IMvigor210	http://research-pub.gene.com/IMvigor210CoreBiologies
OAK and POPLAR cohort	EGAS00001005013	https://ega-archive.org/datasets/EGAD00001007703
NFH cohort	HRA003748	https://ngdc.cncb.ac.cn/gsa-human/browse/HRA003748
Kim et al. cohort	PRJEB25780	https://www.ebi.ac.uk/ena/browser/view/PRJEB25780

**Software and algorithms**		

R version 4.1.1	R project	https://www.r-project.org/
IOBR	Github	https://github.com/IOBR/IOBR
KOBAS-I	KOBAS-I	http://kobas.cbi.pku.edu.cn/kobas3
IKCscore	Github	https://github.com/LiaoWJLab/IKCscore
DESeq2 version 1.36.0	Github	https://github.com/mikelove/DESeq2
Maftools 2.8.0	Github	https://github.com/PoisonAlien/maftools
CIBERSORT	CIBERSORT	https://cibersort.stanford.edu/
GSVA version 1.40.1	Github	https://github.com/rcastelo/GSVA

### Experimental model and study participant details

#### Human specimens

We retrospectively collected tumor tissues from 65 patients with advanced NSCLC before ICBs treatment at the Nanfang Hospital of Southern Medical University. The medical ethics committee of Nanfang Hospital of Southern Medical University approved this study (NFEC-2019-265). The patients provided written informed consent for participation in the study. Detailed information on the individual patients in this study is depicted in Table S1. Clinical responses were evaluated according to the Response Evaluation Criteria in Solid Tumors (RECIST) V.1.1. Patients achieving CR or PR were classified as responders (R), and patients with SD or PD were classified as non-responders (NR). Transcriptomic analysis was performed on tumor specimens from 65 patients with NSCLC. To further validate the predictive power of the IKCscore, we collected 17 specimens for RNA-seq as validation cohort. WES was performed on tumor specimens from 33 patients.

#### Transcriptome sequencing

Total RNA was extracted from tumor specimens of patients with NSCLC using TRIzol reagent. mRNA was purified from total RNA using oligo(dT)-attached magnetic beads and fragmented. According to the manufacturer's protocol, sequencing libraries were constructed using the NEBNext® UltraTM RNA Library Prep Kit for Illumina® (NEB, USA). Subsequently, RNA-seq was performed on an Illumina HiSeq platform, and 150 bp paired-end reads were generated. After filtering for low-quality reads, raw FASTQ files were mapped to the GRCh37 reference genome using the Hisat2 aligner.^42^ The mapped fragments were counted and annotated using HTSeq v0.6.0^43^ and GENCODE v19 annotation files, respectively.

#### Data sources

Multiple independent cohorts were used to validate the predictive value of the model. The GSE135222 cohort included 27 patients with advanced NSCLC treated with anti-PD-1/PD-L1 therapy.^44^ The GSE126044 cohort included 16 NSCLC patients treated with anti-PD-1 therapy.^45^ The OAK and POPLAR cohorts included NSCLC patients treated with atezolizumab,^46^ ant transcriptome data were obtained under accession number EGAS00001005013. The IMvigor210 cohort included patients with metastatic urothelial cancer receiving atezolizumab. Transcriptome and clinical data were downloaded from http://research-pub.gene.com/IMvigor210CoreBiologies. Clinical information, RNA-seq data, and WES data for The Cancer Genome Atlas-Lung adenocarcinoma (TCGA-LUAD) and The Cancer Genome Atlas-Lung squamous cell carcinoma (TCGA-LUSC) cohorts are available from the XENA data portal (http://xena.ucsc.edu/). The Kim et al. cohort was obtained under accession number ENA: ERP107734/SRA: PRJEB25780. The TCR diversity profiling data was available in Bagaev et al. study.^30^

### Method details

#### IKCscore evaluation

We incorporated our IKCscore methodology into an open-source R package, IKCscore (https://github.com/LiaoWJLab/IKCscore), to predict the treatment response of immunotherapy in patients with NSCLC from bulk transcriptomic data.

#### Tumor microenvironment signature score estimation

The IOBR package (https://iobr.github.io/book/)^47^ collected published methods including CIBERSORT, TIMER, MCP counter, and etc. to decode tumor microenvironment contexture. IOBR integrates 255 published signature gene sets, involving tumor microenvironment, tumor metabolism, m6A, and exosomes, and also enrolls the signature gene sets, containing GO, Kyoto Encyclopedia of Genes and Genomes (KEGG), HALLMARK and REACTOME gene sets obtained from MsigDB.^22^ The R package EaSIeR was used to analyze the cell interaction network.^31^ CIBERSORT was used to quantify the proportions of 22 immune cells 13 to estimate immune cell infiltration. The ESTIMATE algorithm was applied to assess tumor purity using RNA-seq data.^15^ Bagaev et al. provided the TCR profiling data of TCGA datasets.^30^ The TCR diversity data of TCGA was downloaded from the supplementary data.

#### Differentially expressed gene analysis (DEGs)

Differential gene analyses between the responder and non-responder groups were performed using the DESeq2 package.^48^ DEGs were considered for further study with an adjusted p-value <0.05. The adjusted p-value for multiple testing was calculated using the Benjamini-Hochberg correction.

#### Functional and pathway enrichment analysis

GO enrichment analysis and KEGG enrichment analysis was conducted using KOBAS-I (http://kobas.cbi.pku.edu.cn/kobas3).^49^ GO terms were identified using a strict cutoff of p < 0.05.

#### Somatic variant detection

After removing poor-quality data and adaptor sequences, reads were aligned to the reference human genome (hs37d5) using the Burrows-Wheeler Aligner (BWA, version 0.7.12-r1039) tool.^50^ Realignment and recalibration were performed using GATK (version v3.6-0-g89b7209).^51^ Single nucleotide variants and small insertions and deletions were counted using MuTect2 (version v3.6-0-g89b7209)^52^ and realDcaller (version 1.5.2, developed in-house).^53^ Based on the above calling results, hotspot variations were evaluated using NChot (version 2.5.9, developed in-house). Somatic copy number alterations were analyzed using CONTRA (version 2.0.8).^54^ Finally, all candidate variants were manually verified using Integrative Genomics Viewer.

#### Immunohistochemistry

Paraffin-embedded slices (4 μm) were dried at 62°C for 1 h. Subsequently, the slices were deparaffinized by xylene and soaked in gradient reduced concentration ethyl alcohol for 3–5 min in order. After antigen retrieval and bovine serum albumin block, the slices were incubated overnight at 4°C with primary antibodies. Next day, secondary antibody incubation was conducted. After 3ʹ3-diaminobenzidine tetrahydrochloride and hematoxylin staining, slices were dehydrated by increasing concentrations of ethanol and xylene. The following antibodies were used for the study: anti-CD3 (Cat# ab16669, Abacm), anti-CD8 (Cat# ab93278, Abcam).

#### WES analysis

The mutation MAF files of TCGA-LUAD and TCGA-LUSC were obtained using TCGAbiolinks,^55^ and the mutation status was inferred from the MAF files. The mutation landscape of NSCLC patients treated with anti-PD-(L)1 treatment was depicted using OncoPrint with R package maftools. The TMB of patients in the NFH cohort was evaluated using R package maftools. The Wilcoxon test was conducted to determine the significance of the IKCscore in gene mutation status (wild-type or mutated).

### Quantification and statistical analysis

The normality of the variables was examined using the Shapiro-Wilk normality test. Significant differences between two groups were determined using unpaired two-tailed t-tests for normally distributed variables, and the Wilcoxon test for non-normally distributed variables; for comparisons between more than two groups, Kruskal-Wallis and one-way analysis of variance tests were used for non-parametric and parametric methods, respectively. Correlation analysis was performed using the Spearman and distance correlation analyses. The Χ^2^ and two-sided Fisher’s exact tests were used to analyze contingency tables. The best cutoff values for the continuous signature score in the survival analysis were evaluated using the R package survminer. Progression-free survival was assessed using the Kaplan–Meier method, and the log-rank test was used to compare survival. The hazard ratios (HRs) were calculated using the univariate and multivariate Cox proportional hazards regression model. A ROC curve was created using the pROC package, and the AUC was used to examine the sensitivity and specificity of the signature scores. The graphical abstract was drawn by Figdraw with ID: YPART00bbf. Statistical analyses were conducted using R software V.4.1.1. All p-values were two-sided, and p-values < 0.05 were considered statistically significant. ns p>= 0.05, ∗ p < 0.05, ∗∗p < 0.01, ∗∗∗p < 0.001. The statistical details of experiments were indicated in each of the figure legend.
