# Tumor microenvironment assessment-based signatures for predicting response to immunotherapy in non-small cell lung cancer

**DOI:** 10.1016/j.isci.2026.114872

**Published:** 2026-02-10

**Authors:** Jiani Wu, Yuanyuan Wang, Zhenhua Huang, Jingjing Wu, Huiying Sun, Rui Zhou, Wenjun Qiu, Zilan Ye, Yiran Fang, Xiatong Huang, Jianhua Wu, Jianping Bin, Yulin Liao, Min Shi, Jiguang Wang, Wangjun Liao, Dongqiang Zeng

## Main text

(iScience *27*, 111340; December 20, 2024)

The *iScience* editorial team is issuing this editorial expression of concern for this article. The concern is that the raw data deposited as dataset HRA003748 remain under controlled access. The authors have stated that the data support the published study; however, due to the inability to access the data, this can’t be verified.

Despite repeated requests since October 2025, the authors have not provided any evidence that the dataset exists and will be accessible to any reader requesting access, nor have they supplied the required documentation of their communications with the repository (China National Center For Bioinformation, Genome Sequence Archive for Human [GSA for Human]), which controls the access of the data. The authors have not responded to the journal since October 31, 2025. To date, no access approvals are being granted, and the dataset remains inaccessible for the research community.

Given the extended inaccessibility of the data and the lack of communication and contradictions from the authors, the editors are expressing concern over the actual existence of the claimed data. Together with the research integrity team, the editors deem it necessary to alert readers to the unavailability of the data supporting this article.

The authors have been instructed to make the dataset publicly available within 6 months of this notice. Failure to do so may result in escalation to retraction.

This notice will remain linked to the article while the matter is unresolved.

