# Supplementary material for: Tumor microenvironment assessment-based signatures for predicting response to immunotherapy in non-small cell lung cancer
Source: iScience. 2024 Nov 13;27(12):111340. doi: 10.1016/j.isci.2024.111340 (PMC11647137; doi:10.1016/j.isci.2024.111340)
Supplement: Document S1. Figures S1–S3 and Tables S1 and S2 [file mmc1.pdf]

iScience, Volume *27*

Supplemental information

Tumor microenvironment assessment-based signatures for predicting response to immunotherapy in non-small cell lung cancer

Jiani Wu, Yuanyuan Wang, Zhenhua Huang, Jingjing Wu, Huiying Sun, Rui Zhou, Wenjun Qiu, Zilan Ye, Yiran Fang, Xiatong Huang, Jianhua Wu, Jianping Bin, Yulin Liao, Min Shi, Jiguang Wang, Wangjun Liao, and Dongqiang Zeng

Supplementary Materials

Figure S1

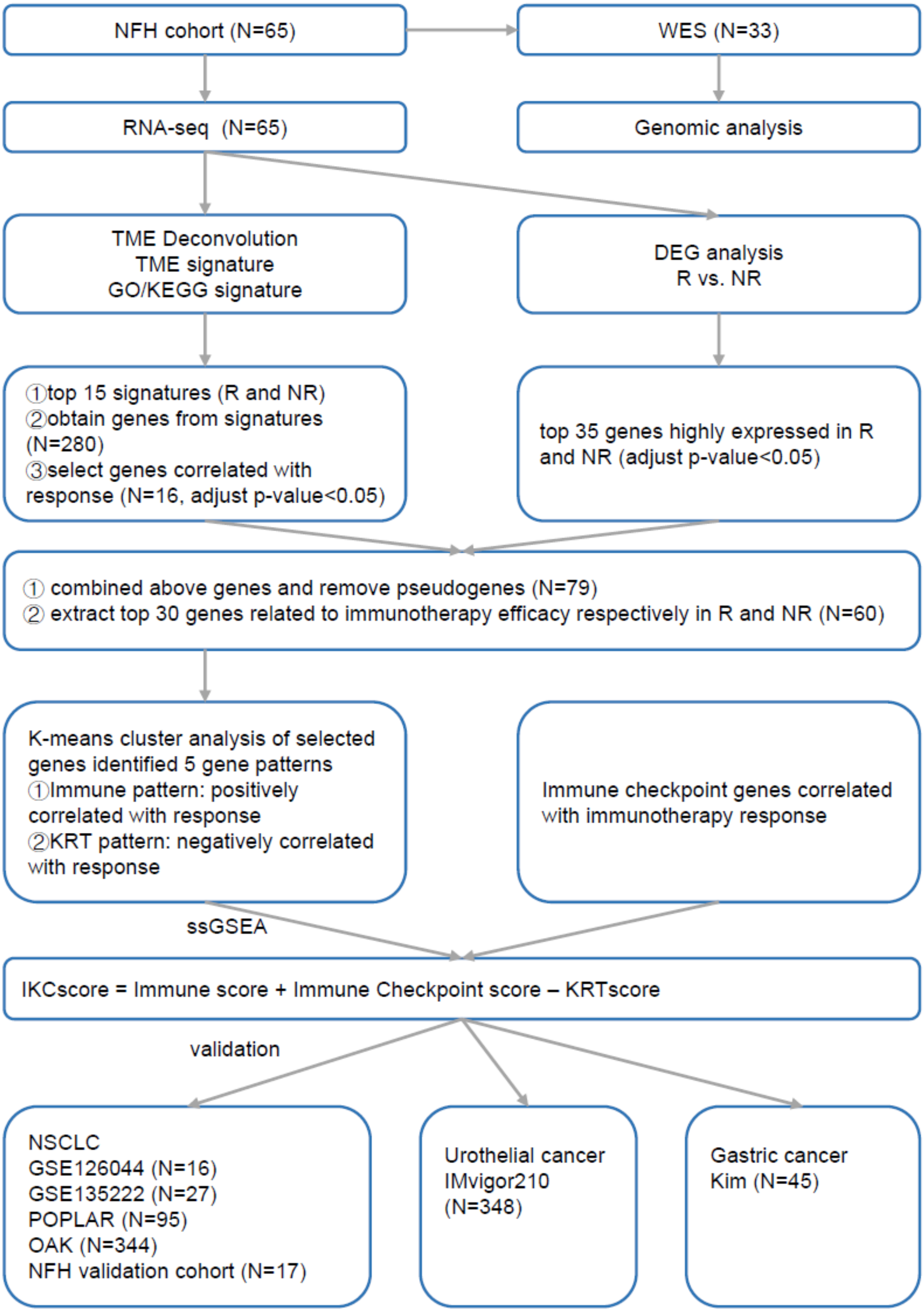

Figure S1 IKCscore construction flowchart, related to Figure 1.

The flowchart shows the IKCscore construction process including signature selection, gene selection and calculation.

Figure S2

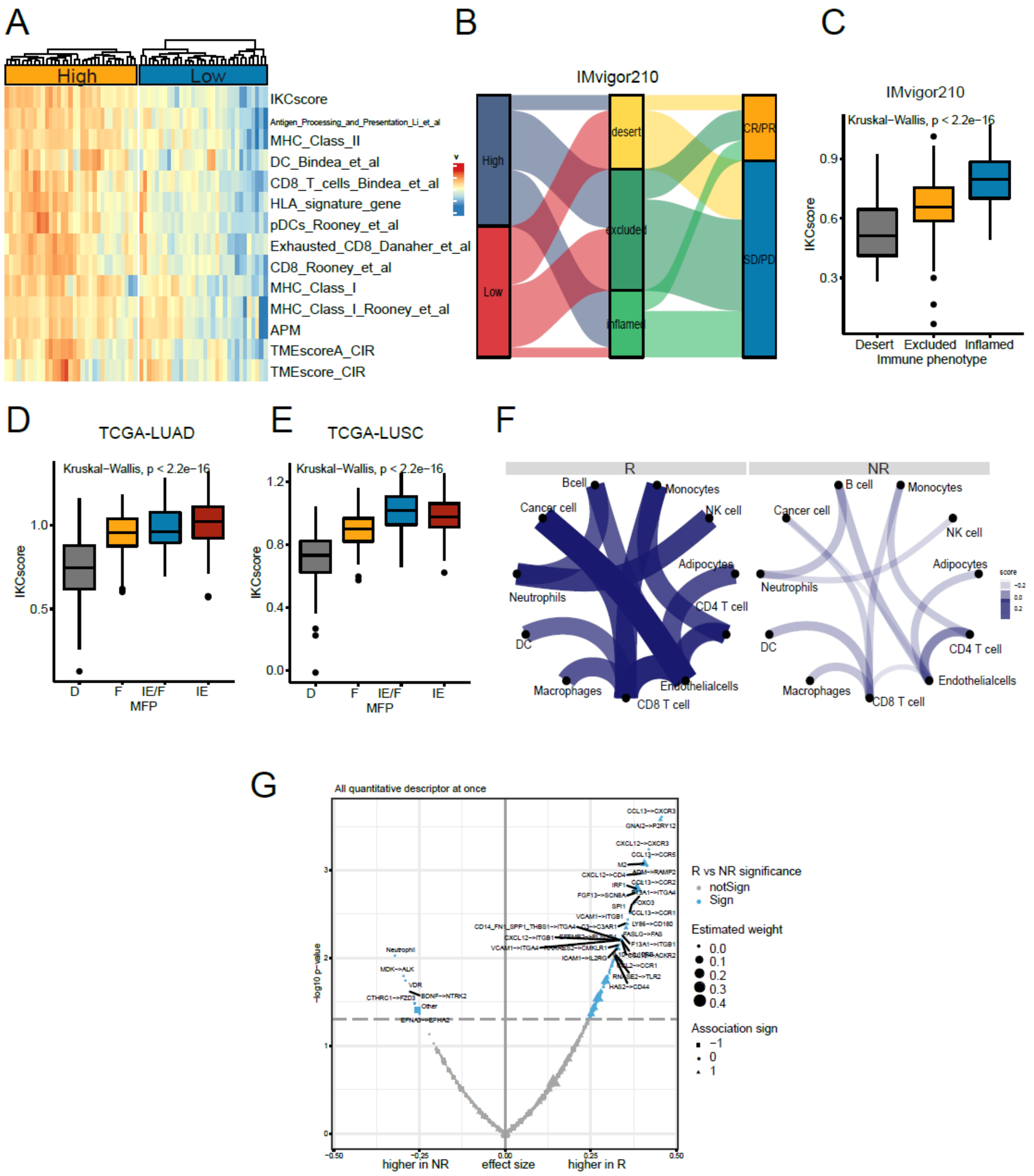

Figure S2 TME landscape associated with ICBs responses, related to Figure 5.

(A) The heatmap illustrated immune pathways in high and low IKCscore tumors. (B) Alluvial diagram of high and low IKCscore groups with different TME subtypes (desert, excluded, and inflamed), and clinical response in IMvigor210 cohort. (C) IKCscore predominantly elevated in inflamed immune subtypes in the IMvigor210 cohort (Kruskal−Wallis, $p < 2.2e{-16}$). (D-E) Boxplot showed that IKCscore mainly increased in the IE subtype, while the lowest IKCscore was observed in the D subtype in TCGA-LUAD (D) and TCGA-LUSC (E). (F) The cell interaction network in responders (R) and non-responders (NR). (G) The volcano plot revealed the ligand-receptor pairs, transcription factors, and other factors related to ICBs responses. IE, immune-enriched, non-fibrotic; IE/F, immune-enriched/fibrotic; F, fibrotic; D, immune-depleted.

Figure S3

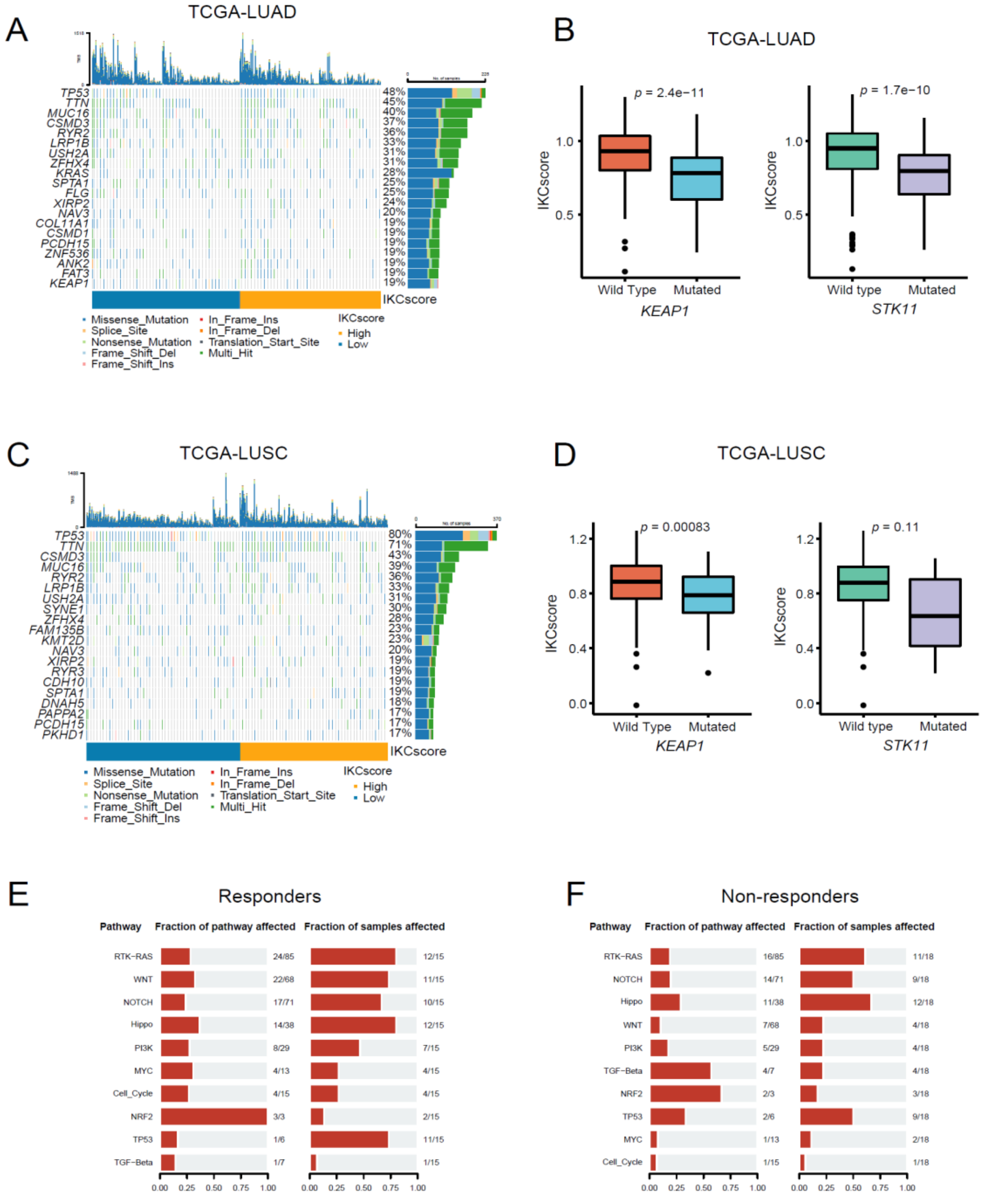

Figure S3 Mutational features associated with IKCscore and responses to immunotherapy, related to Figure 6.

(A) The oncoplot of the top 20 mutated genes in high and low IKCscore groups in the TCGA-LUAD cohort. (B) KEAP1 and STK11 mutations were significantly correlated with lower IKCscore compared with wild type (Wilcoxon test, p = 2.4e-11, p = 1.7e-10, respectively). (C) The oncoplot of top 20 mutated genes in high and low IKCscore groups in TCGA-LUSC cohort. (D) KEAP1 and STK11 mutations were significantly correlated with lower IKCscore compared with wild type (Wilcoxon test, p = 0.00083, p = 0.11, respectively). (E-F) Mutant pathway enrichment in responders (E) and non-responders (F).

Table S1

Patient_ID	Response	Age	Tumor_types	Sex	Treatment
Patient_1	NR	41	Others	M	Combination
Patient_2	NR	43	SQC	M	Combination
Patient_3	R	56	ADC	M	Combination
Patient_4	NR	65	ADC	M	Combination
Patient_5	NR	51	Others	F	Combination
Patient_6	NR	63	ADC	M	Monotherapy
Patient_7	NR	51	ADC	M	Combination
Patient_8	NR	68	ADC	M	Combination
Patient_9	NR	63	Others	M	Combination
Patient_10	NR	63	SQC	M	Combination
Patient_11	NR	39	SQC	F	Combination
Patient_12	R	74	SQC	M	Combination
Patient_13	NR	59	ADC	M	Combination
Patient_14	NR	46	ADC	M	Combination
Patient_15	NR	68	Others	M	Combination
Patient_16	NR	53	ADC	M	Combination
Patient_17	R	52	SQC	M	Combination
Patient_18	R	56	ADC	M	Combination
Patient_19	NR	49	ADC	M	Combination
Patient_20	NR	57	ADC	M	Combination
Patient_21	R	31	SQC	F	Combination
Patient_22	NR	68	SQC	M	Monotherapy
Patient_23	NR	55	Others	F	Combination
Patient_24	NR	59	SQC	M	Monotherapy
Patient_25	NR	53	ADC	F	Monotherapy
Patient_26	R	54	ADC	M	Monotherapy
Patient_27	R	64	Others	M	Monotherapy
Patient_28	NR	58	SQC	M	Monotherapy
Patient_29	R	54	ADC	M	Monotherapy
Patient_30	R	65	ADC	M	Combination
Patient_31	R	76	ADC	M	Combination
Patient_32	R	50	ADC	F	Combination
Patient_33	NR	81	SQC	F	Monotherapy
Patient_34	R	50	ADC	F	Monotherapy
Patient_35	NR	52	Others	F	Combination
Patient_36	NR	56	ADC	M	Combination
Patient_37	NR	58	ADC	M	Monotherapy
Patient_38	R	72	ADC	M	Combination
Patient_39	NR	54	SQC	F	Combination
Patient_40	R	75	ADC	M	Monotherapy
Patient_41	R	62	SQC	M	Combination
Patient_42	NR	66	ADC	F	Combination
Patient_43	R	52	ADC	M	Combination
Patient_44	R	54	ADC	M	Combination
Patient_45	R	69	SQC	M	Combination
Patient_46	R	54	SQC	M	Combination
Patient_47	R	59	ADC	M	Combination
Patient_48	NR	55	ADC	M	Combination
Patient_49	NR	63	ADC	M	Combination
Patient_50	NR	66	SQC	M	Combination
Patient_51	R	56	Others	M	Combination
Patient_52	NR	56	ADC	M	Combination
Patient_53	R	58	ADC	M	Combination
Patient_54	NR	59	Others	M	Combination
Patient_55	R	78	ADC	M	Monotherapy
Patient_56	NR	69	ADC	M	Combination
Patient_57	NR	48	ADC	M	Combination
Patient_58	NR	75	SQC	M	Combination
Patient_59	R	69	SQC	M	Combination
Patient_60	NR	59	ADC	M	Combination
Patient_61	R	68	ADC	M	Combination
Patient_62	NR	66	SQC	M	Combination
Patient_63	NR	58	SQC	F	Monotherapy
Patient_64	NR	62	ADC	F	Combination
Patient_65	NR	57	SQC	M	Combination

Table S1 Patient characteristics of the advanced NFH cohort treated with anti-PD-1 immunotherapy, related to Figure 1 and Table 1.

Table S2

Immune score	KRT score	Immune Checkpoint score
MS4A6A	KRTAP5-5	CD274
C1QC	KCNC1	PDCD1LG2
HLA-DQB1	ADAM30	CTLA4
RAMP2	CELF3	PDCD1
MATN3	PNMA5	LAG3
CDK15	POU3F2	HAVCR2
WNT2	UNC80	TIGIT
FOLR2	TMEM132D	
HPGD	ATP2B3	
KDR	CPLX2	
LGMN	ZFP42	
PTGER2	INA	
F13A1	KRTAP5-1	
SLCO2B1	GP2	
TMEM100	SCGN	
	ST18	
	BLOC1S5-TXNDC5	
	FMN2	
	ANO3	

Table S2 Gene lists of immune, KRT, and Immune Checkpoint patterns, related to Figure 1.
